# The Role of Host Cholesterol During Flavivirus Infection

**DOI:** 10.3389/fcimb.2018.00388

**Published:** 2018-11-02

**Authors:** Juan Fidel Osuna-Ramos, José Manuel Reyes-Ruiz, Rosa Maria del Ángel

**Affiliations:** Department of Infectomics and Molecular Pathogenesis, Center for Research and Advanced Studies (CINVESTAV-IPN), Ciudad de Mexico, Mexico

**Keywords:** flavivirus, arbovirus infection, host cholesterol, IFN response, lipid-lowering drugs

## Abstract

In recent years the emergence and resurgence of arboviruses have generated a global health alert. Among arboviruses, Dengue (DENV), Zika (ZIKV), Yellow Fever (YFV), and West Nile (WNV) virus, belong to the genus *Flavivirus*, cause high viremia and occasionally fatal clinical disease in humans. Given the genetic austerity of the virus, they depend on cellular factors and organelles to complete its replication. One of the cellular components required for flavivirus infection is cholesterol. Cholesterol is an abundant lipid in biomembranes of eukaryotes cells and is necessary to maintain the cellular homeostasis. Recently, it has been reported, that cholesterol is fundamental during flavivirus infection in both mammal and insect vector models. During infection with DENV, ZIKV, YFV, and WNV the modulation of levels of host-cholesterol facilitates viral entry, replicative complexes formation, assembly, egress, and control of the interferon type I response. This modulation involves changes in cholesterol uptake with the concomitant regulation of cholesterol receptors as well as changes in cholesterol synthesis related to important modifications in cellular metabolism pathways. In view of the flavivirus dependence of cholesterol and the lack of an effective anti-flaviviral treatment, this cellular lipid has been proposed as a therapeutic target to treat infection using FDA-approved cholesterol-lowering drugs. This review aims to address the dependence of cholesterol by flaviviruses as well as the basis for anti flaviviral therapy using drugs which target is cholesterol synthesis or uptake.

## Introduction

Viral infections transmitted by mosquitoes, such as those caused by the flaviviruses dengue (DENV), yellow fever virus (YFV), West Nile virus (WNV), and Zika virus (ZIKV) represent important health challenges. In the last years, the emergence or reemergence of different arboviruses have generated a global health alert. In this sense, DENV continues to increase in tropical and subtropical regions of the world, whereas outbreaks of YFV in humans have been reported in Angola and some countries in South America. Moreover, the emergence of ZIKV, which is strongly associated with microcephaly in newborns, Guillain-Barre syndrome in adults and the ability to be transmitted sexually and through trans placental route, as well as the neurotropic behavior of WNV; making these viruses a latent threat to global health (Barrett, 2017; Valderrama et al., 2017; Salles et al., 2018; Silva et al., 2018; Talero-Gutiérrez et al., 2018). Although, there is an approved and effective vaccine for YFV, an effective vaccine or treatment for DENV and ZIKV have not been achieved yet (Barrett, 2017; Silva et al., 2018). Therefore, it is urgent to develop an effective therapy against these viruses. Flaviviruses use different strategies to replicate and to evade immune response; one of them is the inhibition of interferon (IFN) response mediated by at least, in the case of DENV, of the non-structural proteins NS2A, NS4A, NS4B, and NS5 which target the signal transducer and activator of transcription proteins 1 and 2 (STAT1 and STAT2) (Morrison et al., 2012). A second mechanism to inhibit immune response is common for many RNA viruses and is the use of the non-structural viral proteins to modify the endoplasmic reticulum (ER) membranes to generate partially isolated compartments known as replication complexes (RC) where the new viral particles are replicated and formed (Welsch et al., 2009; Neufeldt et al., 2018). The RC as replicative organelles are fundamental because they create a barrier that minimizes the detection of double-stranded RNA or 5′-phosphorylated RNA in the cytoplasm (Neufeldt et al., 2018). Interestingly, the formation of the RC requires fatty acids, cholesterol, glycerophospholipids, phospholipids, and sphingolipids (mainly ceramides) (Heaton et al., 2010; Neufeldt et al., 2018). To this regard, the NS3 protein sequesters the fatty acids synthase (FASN) to the RC, and viral infection increases the cholesterol uptake and cholesterol synthesis (Heaton et al., 2010; Soto-Acosta et al., 2013, 2017). A wide variety of lipids (fatty acids, glycerolipids, glycerophospholipids, sphingolipids, sterols, prenolic lipids, saccharolipids, and polychaetes) can be found in cell membranes (Fahy et al., 2005). Cholesterol is an abundant lipid in biomembranes of eukaryotes cells and is essential for adequate cellular functioning (Simons and Ikonen, 2000; Crane and Tamm, 2004; Fernández et al., 2004). Cholesterol levels in the cells are controlled by biosynthesis, efflux from cells, and uptake (Simons and Ikonen, 2000). Some cholesterol uptake receptors that participate during DENV infection are the low-density lipoprotein receptor (LDLr) and the scavenger receptor class B type I (SR-BI) (Betters and Yu, 2010; Li et al., 2013; Soto-Acosta et al., 2013), while the increase in cholesterol synthesis during DENV infection is mediated by the increase in the activity of the 3-hydroxy-3-methyl-glutaryl-coenzyme A reductase (HMGCR, limiting enzyme in the cholesterol synthesis pathway) being the activation of the HMGCR a consequence of the inhibition of the molecule considered as the cellular metabolism controller, the AMP-dependent kinase (AMPK) (Ikonen, 2008; Cerqueira et al., 2016).

**Figure 1:**
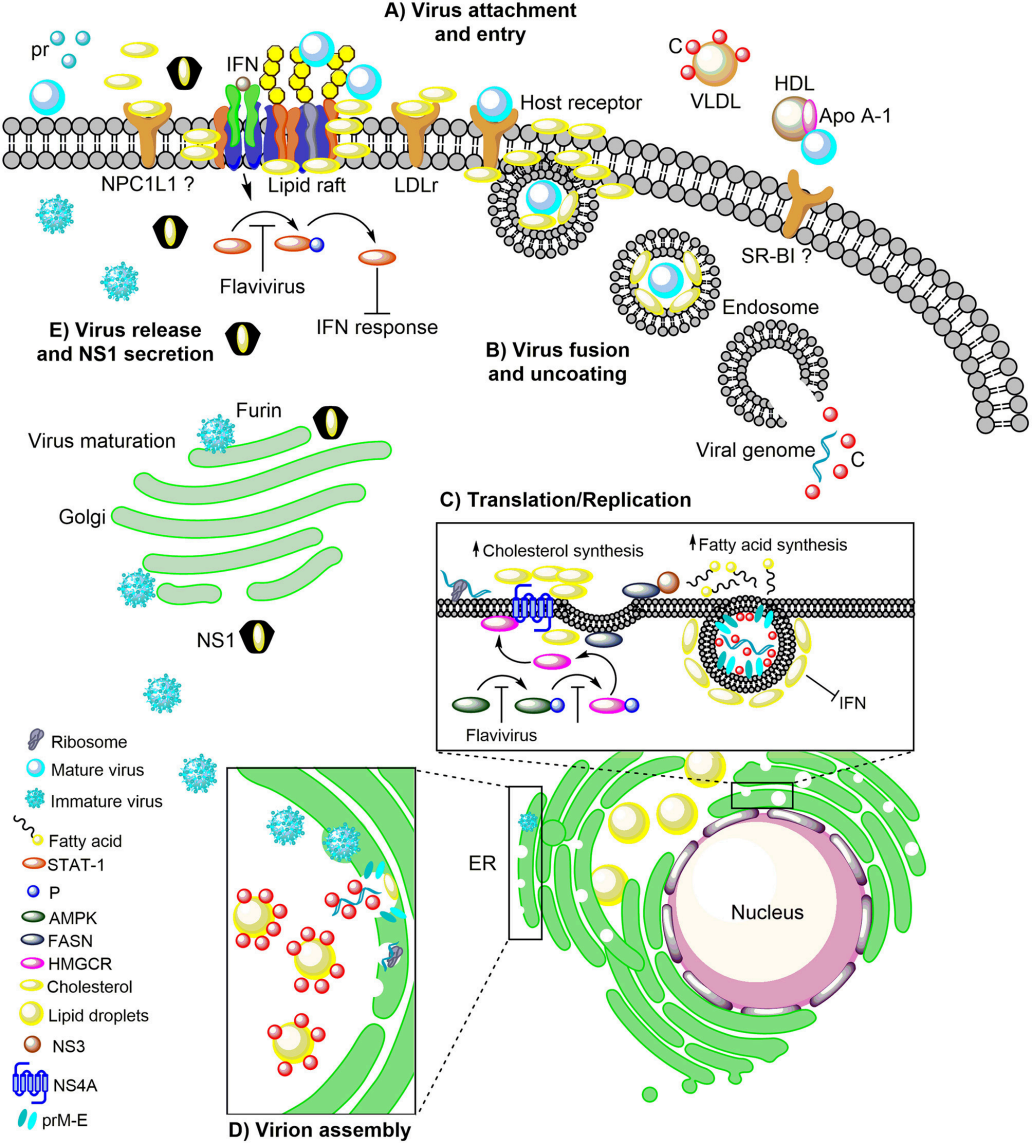
The role of host cholesterol during the replicative cycle of the flaviviruses. **(A)** Upon attachment to the cell surface, virus particles bind to the distinct receptors found in lipid rafts, cholesterol-rich plasma membrane microdomains, to trigger clathrin-dependent endocytosis. Immediately after attachment and entry, there is an increase in cholesterol levels, which correlates with an augment of the LDLr, on the surface of the infected cell. The importance of NPC1L1 and SR-BI in flaviviral infection has been suggested. **(B)** Following, the low pH in the endocytic vesicle induces fusion between endosomal and viral membranes. **(C)** The viral RNA into the cytoplasm is transported to the ER where it is translated into the three structural proteins (C, prM, and E) and the seven non-structural proteins (NS1, NS2A, NS2B, NS3, NS4A, NS4B, and NS5). RNA replication occurs in NS4A protein-induced membrane invaginations called RC, where the fatty acids and cholesterol synthesis take place. In this event, NS3 recruit the FASN to the ER increasing its activity. Additionally, the flaviviral infection inhibits the AMPK activity inducing a reduction in the phosphorylation levels of HMGCR, which leads to an increase in its activity and cholesterol synthesis. This mechanism occurs in the ER where the FASN and HMGCR enzymes are found associated with the viral proteins NS3 and NS4A. **(D)** The flavivirus-induced membranes are rich in cholesterol and serve as a scaffold for the assembly of the viral progeny. In the ER the C protein is accumulated on the LDs for the packaging of the viral genome and the nucleocapsid formation. Later the nucleocapsids bud in the ER to complete the assembly of immature virions which are transported to the Golgi complex for its maturation. **(E)** The maturation of viral particles occurs along the secretory pathway where the low pH triggers rearrangements in the structural proteins (prM) to allow the proteolytic cleavage of the pr peptide by furin protease. Finally, mature (infectious) virions are released from the cell by exocytosis. The DENV NS1 protein is also secreted and has a peculiar three-dimensional fold as hexamer that forms a lipoprotein particle with an open-barrel protein shell and a central channel rich in lipids (cholesterol), reminiscent of the composition of high-density lipoprotein. Furthermore, NS1 play an essential role in the pathogenesis, viral replication, and immune evasion. In this sense, the flaviviruses block the IFN signal transduction pathway either by inhibiting of the phosphorylation of STAT-1 which is found in the cholesterol-rich microdomains (lipid rafts) or hijacking cell elements to form their cholesterol-rich RC that avoid the recognition of viral component for the PRR. LDLr, Low-density lipoprotein receptor; NPC1L1, Niemann Pick C1-like 1 receptor; VLDL, Very low-density lipoprotein; DENV, Dengue virus; Apo A-1, Apolipoprotein A-1; HDL, High-density lipoprotein; SR-BI, Scavenger receptor class B type I; ER, Endoplasmic reticulum; RC, Replicative complexes; FASN, Fatty acid synthase; AMPK, Adenosine Monophosphate-activated Protein Kinase; HMGCR, 3-hydroxy-3-methyl-glutaryl-coenzyme A reductase; SREBP, Sterol regulatory element binding protein; LDs, Lipid droplets; IFN, Interferon; STAT-1, Signal transducer and activator of transcription 1; P, Phosphorylation; PRR, Pattern recognition receptors.

On the other hand, there is a close correlation between cholesterol levels and type 1 IFN response. High levels of cholesterol induce a poor IFN response (Liu et al., 2013). Thus, the reduction in cholesterol levels during flavivirus infection grants an increase in IFN response. Despite how much is known about the role of cholesterol during DENV infection (Rothwell et al., 2009; Puerta-Guardo et al., 2010; Poh et al., 2012; Carro and Damonte, 2013; Soto-Acosta et al., 2013, 2017), its importance during YFV, WNV and ZIKV infection is understudied. Therefore, understanding the importance of host cholesterol during flavivirus infections allow us to design antiviral strategies with cholesterol-lowering drugs such as statins (Rothwell et al., 2009; Martínez-Gutierrez et al. 2011, 2014; Soto-Acosta et al., 2013; Bryan-Marrugo et al., 2016), or with AMPK activators such as metformin (Soto-Acosta et al., 2017; Htun et al., 2018), and the nordihydroguaiaretic acid (NDGA) compound (Soto-Acosta et al., 2014; Merino-Ramos et al., 2017); which reduce cholesterol synthesis as well as with drugs that inhibit cholesterol uptake such as ezetimibe. This review is mainly focused on the recent findings that demonstrate how the mosquito-borne flaviviruses take advantage of host-cholesterol to complete their life cycle (Figure 1) and host-directed antiviral (HDA) therapy strategies for flavivirus inhibition.

## The role of host cholesterol during the flavivirus life cycle

### Host cholesterol in flavivirus entry

The arboviruses of the Flavivirus genus, Flaviviridae family members, as enveloped viruses, have a major and conserved E glycoprotein, which is composed of ninety dimers arranged with a quasi-icosahedral symmetry on the viral membrane (Nybakken et al., 2006; Li et al., 2008; Dai et al., 2016). This protein is highly conserved in the Flavivirus genus and is involved in viral attachment to the mosquito and mammalian host cells. The first step in viral infections is the binding and entry process. In this step, the viral particles have to bind to specific molecules on the cell surface such as receptors and coreceptors to enter into the host cell (Figure 1A). Since several of the attachment and receptor molecules described for viruses are present in lipid rafts (Takahashi and Suzuki, 2009), lipids and cholesterol-rich plasma membrane microdomains that are essential during entry of DENV (Lee et al., 2008; Puerta-Guardo et al., 2010; Soto-Acosta et al., 2013; García Cordero et al., 2014; Reyes-del Valle et al., 2014; Diwaker et al., 2015), and WNV (Medigeshi et al., 2008) (Figure 1A). The use of cholesterol-depleting drugs such as methyl-β-cyclodextrin (MβCD) or filipin, which binds to cellular cholesterol forming complexes and avoiding the formation of lipid rafts before viral entry. It has been demonstrated to induce an antiviral effect for DENV and WNV infection, which made it possible to confirm the importance of lipid rafts during entry for both viruses (Lee et al., 2008; Medigeshi et al., 2008; Rothwell et al., 2009). To the contrary, cholesterol and lipid rafts are not essential during the entry in the mosquito cell line C6/36 (*Aedes albopictus*) infected with DENV (Mosso et al., 2008) and WNV (Chu et al., 2006). Although insects cannot synthesize cholesterol *de novo*, they can take it to synthesize cell membranes and hormones (CLAYTON et al., 1962; CLAYTON, 1964; Krebs and Lan, 2003). Recently, cholesterol molecules were detected during the early stages of DENV replication in the middle intestine of *Aedes aegypti* mosquitoes (Chotiwan et al., 2018). However, the specific role of the cholesterol in this organ in mosquito-virus interaction remains unexplored.

The cholesterol dependence of DENV entry and post-entry steps have been observed in several mammalian cell lines (Lee et al., 2008; Soto-Acosta et al., 2013; Martínez-Gutierrez et al., 2014), it does not seem to be a general event, because it has been described that cholesterol is not required for Vero (green monkey epithelial kidney cell line) (Acosta et al., 2009; Carro and Damonte, 2013), HepG2 (Hepatocarcinoma cell line) and ECV304 cells entry (human endothelial cell line) (Rothwell et al., 2009). In addition, it has been described that the molecular tweezer CLR01, a small molecule that previously has been shown to inactivate some viruses through a selective interaction with the host-membrane-derived lipid bilayer of the viral envelope, inhibit EBOV (ebola virus) and ZIKV infection (Röcker et al., 2018).

Immediately after attachment and entry of the viral particles by clathrin-mediated endocytosis (Chu et al., 2006; Mosso et al., 2008; Acosta et al., 2009), at one and 48 h post-infection, an increase in cholesterol levels is observed in infected cells in mammalian cells (Soto-Acosta et al., 2013). This increment correlates with an increase in the presence of the low-density lipoprotein receptor (LDLr) on the surface of infected cells and with an augment in the cholesterol uptake (Soto-Acosta et al., 2013), indicating that cholesterol is essential during the first few hours of infection. On the other side, has been described that the structural protein C of DENV can interact with very low-density lipoproteins (VLDL) (Faustino et al., 2014). Faustino et al., using atomic force microscopy-based force spectroscopy, dynamic light scattering, nuclear magnetic resonance, and computational approach; demonstrated that dengue viral capsid proteins (C protein) bind to very low density lipoprotein (VLDL) surfaces (Faustino et al., 2014) (Figure 1A). This observation suggests the formation of lipoviroparticles (LVPs) in DENV infection. However, the presence of LVPs has not been observed during *in vivo* DENV infection, and the direct function of LVPs in DENV attachment or entry steps has not been analyzed (Reyes-del Valle et al., 2014). Besides, there is a report where apolipoprotein A-1 (Apo A-1), the main component of high-density lipoprotein (HDL), interact with DENV particles and facilitates viral entry through the scavenger receptor class B type I (SR-BI), the cell receptor for Apo A-I (Li et al., 2013) (Figure 1A). These observations provide evidence on the functional importance of lipoproteins and cholesterol uptake through cholesterol receptors during DENV infection. Moreover, the importance of the intracellular trafficking of cholesterol during the DENV entry has been demonstrated when this traffic is inhibited by the drug U18666A which mimic Niemann-Pick type C disease (hereditary lysosomal storage disease), causing the accumulation of cholesterol and the entrapment of DENV particles in late endosomes and lysosomes, reducing levels of viral genome released into the cytoplasm of treated cells (Poh et al., 2012).

### Host cholesterol in viral fusion

As enveloped viruses, flaviviruses need to be uncoated to release the viral RNA into the cytoplasm (Figure 1B). Uncoating is induced by the low pH environment of the endosomes, where the viral proteins enter into a fusion-active state and initiate the merging of the viral envelope with the endosomal membrane, thereby releasing the viral RNA genome into the cytoplasm (Kaufmann and Rossmann, 2011; Smit et al., 2011). This process requires two steps, the fusion between the viral and endosomal membranes and the uncoating of the protective capsid shell (Rumlová and Ruml, 2018). To analyze the fusion event, the lipid composition of the viral membrane of different viruses has been characterized (Brügger et al., 2006; Kalvodova et al., 2009; Merz et al., 2011; Gerl et al., 2012; Reddy and Sansom, 2016). In the *Flaviviridae* family, an essential role of membrane virion-associated cholesterol has been demonstrated for all serotypes of DENV (Carro and Damonte, 2013). As it has been shown for HCV (Aizaki et al., 2008) (another member of the family *Flaviviridae*, but not an arbovirus), the presence of cholesterol in DENV virions was more crucial for infection (Carro and Damonte, 2013). In this regard, Carro and Damonte showed that after exposure of DENV particles to MβCD a loss of infectivity of the four serotypes, associated with a reduction in the cholesterol content of the virions was observed.

Moreover, the addition of exogenous water-soluble cholesterol or fetal bovine serum did not fully recover the infectivity of virions, except, when a simultaneous incubation with MβCD and serum cholesterol was performed (Carro and Damonte, 2013); this is consistent with others reports in which authors try to restore the cholesterol that has been depleted from viral particles (Sun and Whittaker, 2003; Lee et al., 2008; Desplanques et al., 2010).

Interestingly, when virions were incubated only with exogenous cholesterol, an inactivating effect on particle infectivity was observed. This effect is similar that the one observed by Lee et al., allowing to suggest that excess in cholesterol could induce an increase in envelope rigidity, which in turn would limit the ability of the viral membrane to fuse inhibiting viral entry (Lee et al., 2008). The MβCD removes not only cholesterol but also other components as phospholipids from the membranes. Therefore, the possibility that MβCD alters the composition of other phospholipids in the viral particle has to be considered (Zidovetzki and Levitan, 2007; Carro and Damonte, 2013). On the other hand, in an *in vitro* study, using a liposomal model system, it has been revealed that flaviviruses such as WNV can fuse with these receptor-free artificial lipid membranes, consisting of phosphatidylcholine and phosphatidylethanolamine at low pH, although with low efficiency. However, the addition of cholesterol to the target membranes has a strong promoting effect on the fusion capacity of WNV (Moesker et al., 2010). Other studies of virus-liposome co-flotation have indicated that cholesterol stimulates the interaction of glycoprotein E with lipid membranes (Stiasny et al., 2003; Umashankar et al., 2008), confirming the importance of cholesterol and specifically of the 3-hydroxyl group of cholesterol for this function. In contrast, the glycoprotein E of alphaviruses does not appear to interact directly with cholesterol in the target membrane (Umashankar et al., 2008). These observations suggest that cholesterol could be required to induce changes in the fluidity of the viral membrane or changes in the physicochemical properties of the membrane required during viral and endosomal membranes fusion (Smit et al., 2011). In an attempt to analyze the molecular composition of the flavivirus envelope, the composition of the lipid envelope of WNV virions was studied. Authors describe that the viral membrane is enriched in sphingolipids (sphingomyelin) and contains reduced levels of phosphatidylcholine (Martín-Acebes et al., 2014). Interestingly, by constructing a computational model of the DENV envelope, in which the known structure of the DENV membrane proteins was combined with a lipidic bilayer model. The authors found that despite the absence of cholesterol in the envelope, the virion presented a biophysical robustness that coincided with the level of cholesterol in the membrane of the influenza A virus (Reddy and Sansom, 2016). Thus, more studies have to be performed to determine the contribution of cholesterol to the biophysical properties of the viral particle. It is evident that the viral membrane is acquired in the ER (Welsch et al., 2009). Thus, the properties of the envelope may depend on the host from which the virions are released.

### Host cholesterol in viral translation/replication

After internalization and uncoating (Samsa et al., 2009), the viral RNA is translated into a polyprotein which is cleaved into structural and non-structural proteins. The viral proteins synthesized such as the NS4A protein induces the ER-membrane remodeling to form membrane curvatures (Roosendaal et al., 2006; Miller et al., 2007). Consequently, the extensive membrane curvature could reduce the effective surface area and functionality of the ER (Heaton et al., 2010). Moreover, these invaginations of the ER leads to the redistribution of the FASN (Heaton et al., 2010) and the cholesterol-synthesizing enzyme, the HMGCR (Soto-Acosta et al., 2017) required for the synthesis of mevalonate, a precursor of the cholesterol, which is upregulated in response to cholesterol depletion (Goldstein and Brown, 1990). It is suggested that this mechanism occurs when NS3 recruit to FASN in the ER where the N-terminal 180 amino acids of NS3 have interaction with FASN and this association increase the FASN activity and the fatty acid biosynthesis to augment the lipid biogenesis to form the replicative complexes (Heaton et al., 2010) (Figure 1C). Moreover, the HMGCR is redistributed from the outer membrane of the nuclear envelope, where it is synthesized and localized (Pathak et al., 1986) to sites of viral replication (Peña and Harris, 2012; Soto-Acosta et al., 2017). Interestingly, although the induction of HMGCR expression during DENV infection does not occur, an increase in the activity of the enzyme due to a reduction of its phosphorylation levels was observed (Soto-Acosta et al., 2013). However, the action of viral proteins NS3 and NS4A on the HMGCR activity is unknown and the mechanism by which NS3 stimulates FASN-specific activity needs to be investigated further (Heaton et al., 2010). In summary, the presence and the increased activity of the FASN and HMGCR enzymes in the ER and their association with viral proteins such as NS3 and NS4A increase the *de novo* synthesis of fatty acids and cholesterol (Heaton et al., 2010; Soto-Acosta et al., 2017) (Figure 1C). This event induces the formation of the RC that comprise membrane packets (Vp), double-membrane vesicles (Ve), tubular structures (T), and convoluted membranes (CM) (Welsch et al., 2009; Junjhon et al., 2014). Inside of the Ve where the NS1, NS3, and NS5 proteins and dsRNA are localized, the RNA replication occurs (Welsch et al., 2009; Junjhon et al., 2014).

The complex set of membranes required for the viral replication is host-dependent (Perera et al., 2012). Consequently, the structures of convoluted membranes (CM) are found in DENV-infected mammalian cells (Welsch et al., 2009), but they are not induced in DENV-infected C6/36 cells (Junjhon et al., 2014; Reyes-Ruiz et al., 2018). Since the cholesterol contributes to the stability of subcellular structures, it is possible that the absence of CM could be related with the fact that the mosquitoes are cholesterol auxotrophs (CLAYTON, 1964; Rawson, 2003) and the amount of this molecule is lower in mosquito cells compared to mammalian cells. Under experimental conditions, the bovine serum from the culture medium provides the cholesterol that mosquito cells required (CLAYTON, 1964; Krebs and Lan, 2003). Considering that ~1% of unesterified cholesterol is found in the ER (Lange et al., 1999; Liscum and Munn, 1999), the cholesterol requirement during viral replication is high. Thus, viruses directly manipulate the host cell pathways involved in the uptake and biosynthesis of cholesterol to increase the levels. When the cholesterol is low in the ER, the cholesterol sensor SREBP (Sterol regulatory element binding protein)-SCAP (SREP cleavage activating protein) complex is transported to the Golgi apparatus where the cytoplasmic domain of SREBP is cleaved and the protein is translocated into the nucleus acting as a transcription factor to induce HMGCR and LDLr gene transcription (Vallett et al., 1996). It has been reported that upregulation of enzymes involved in the intermediate steps of cholesterol biosynthesis such as HMGCR and mevalonate diphospho decarboxylase (MVD) lead to higher cholesterol levels in the ER which favor the replication of WNV (Mackenzie et al., 2007) and DENV (Rothwell et al., 2009; Soto-Acosta et al., 2013, 2017). On the other hand, the modulation of exogenous cholesterol uptake by critical proteins such as the LDLr plays an essential role in flavivirus replication (Poh et al., 2012; Soto-Acosta et al., 2013). The use of drugs that inhibit cholesterol biosynthesis pathway can alter the RC formation inhibiting virus infection (Mackenzie et al., 2007; Rothwell et al., 2009; Martínez-Gutierrez et al., 2014; Soto-Acosta et al., 2017); demonstrating the essential role of the *de novo* synthesis of cholesterol during viral replication.

In mosquito cells little is known about the role of cholesterol during replication. However, Perera et al. demonstrated that DENV induces changes in the lipids profile of infected mosquito cells (Perera et al., 2012). Unfortunately, their mass spectrometry analysis was unable to successfully separated cholesterol. On the other hand, in *Wolbachia*-infected mosquito cells, it has been reported the upregulation of the apolipoprotein D, the ATP-binding cassette protein A1 (ABCA1), and a homolog of cholesterol transporter NPC2, and the downregulation of the LDLr (Geoghegan et al., 2017). All these proteins are involved in the cholesterol homeostasis. These results are exciting because the mosquitoes are cholesterol auxotrophs (Rawson, 2003) as well as the *Wolbachia* bacteria (Wu et al., 2004). Thus, both depend on and compete for cholesterol (Caragata et al., 2013). This competence for cholesterol is one of the reason that justify the inhibition of viral replication by *Wolbachia* (Moreira et al., 2009; Bian et al., 2010; van den Hurk et al., 2012; Caragata et al., 2016; Dutra et al., 2016; Geoghegan et al., 2017). However, further studies have to be performed to completely elucidate the role of cholesterol in flaviviral replication in mosquito infected cells.

### The importance of host cholesterol in the viral assembly

The flavivirus-induced membrane rearrangements also serve as a scaffold for the assembly of the viral progeny. After uncoating, translation and genome replication, the assembly of the viral particles is carried out in the replication complexes induce in the ER. These assembly sites have a high activity of enzymes such as the cholesterol-synthesizing enzyme, HMGCR and MVD involved in the cholesterol biosynthesis pathway (Mackenzie et al., 2007; Rothwell et al., 2009; Soto-Acosta et al., 2013, 2017), which leads to the replication complexes being rich in cholesterol. The sites for viral assembly and budding have been reported as the membrane packets (Vp), which contain anchored the viral E and prM proteins to their membrane (Junjhon et al., 2014). Although the mosquito lacks the biosynthetic pathways to produce cholesterol, the Vp are present in both, mammalian and mosquito cells (Welsch et al., 2009; Junjhon et al., 2014; Reyes-Ruiz et al., 2018). The first step in the flavivirus assembly is the packaging of one copy of the viral RNA in multiple copies of the capsid (C) protein to form the nucleocapsid (Lindenbach and Rice, 2003). The viral C protein is located in a close proximity of the ER surrounding the structures called lipid droplets (LDs) (Samsa et al., 2009; Carvalho et al., 2012), which are structurally similar to circulating lipoproteins with a core of esterified lipids, where the cholesterol is an essential component (Ducharme and Bickel, 2008). The viral RNA recruitment process by the C protein to form the nucleocapsid is still unclear. However, it has been suggested that the C protein or the C protein-RNA complex is accumulated on the LDs during infection to be then mobilized to viral assembly sites (Samsa et al., 2009; Carvalho et al., 2012) (Figure 1D). Interestingly, the accumulation of C protein on LD occurs in both infected mammalian and mosquito cells, which suggest that the LD are organelles with a conserved function in the viral replication in different hosts (Samsa et al., 2009) and crucial for viral assembly. Once that the nucleocapsid is formed, this is enveloped by a lipid membrane (Kuhn et al., 2002), from the ER where are found the prM-E heterodimers to complete the assembly of immature virion and then the viral particle is transported to the Golgi complex for its maturation (Mukhopadhyay et al., 2005; Yu et al., 2008; Welsch et al., 2009).

### Role of cholesterol in viral release and NS1 secretion

The last step of the flaviviruses replicative cycle is the release of viral particles by exocytosis (Barrows et al., 2018). The viral particles to become infectious have to maturate by the cleavage of prM protein by the host protease furin (Pierson and Diamond, 2012) (Figure 1E). In this step, the participation of cholesterol has not been described.

On the other hand, during flavivirus infection, the non-structural protein 1 (NS1) is secreted (Scaturro et al., 2015). The NS1 protein is one of the most enigmatic proteins of flaviviruses because it is the only protein that is secreted, playing essential roles in immune evasion, pathogenesis and viral replication (Scaturro et al., 2015). The reported crystal structure of the secreted DENV NS1 protein revealed its peculiar three-dimensional fold as hexamer that forms a lipoprotein particle with an open-barrel protein shell and a central channel rich in lipids, reminiscent of the composition of high-density lipoprotein (Gutsche et al., 2011). NS1-associated lipid species include triglycerides, as well as cholesterol and phospholipid esters, a composition that evokes plasma lipoproteins in humans involved in vascular homeostasis. Thus, the discovery that DENV NS1 carries lipids could have critical pathophysiological implications during the disease (Gutsche et al., 2011) and supports the idea that cholesterol is required for the secretion of NS1 (Figure 1E).

## Modulation of the host-immune response mediated by cholesterol

All flaviviruses share similar replication strategies manipulating host cell functions for successful infection. One of these strategies is the evasion of the antiviral responses in both, the hematophagous invertebrate vectors and vertebrate hosts (Ciota and Kramer, 2010; Coffey et al., 2013). The type I interferon (IFN) response constitutes the first line of defense for the early control of viral infections (Muñoz-Jordán et al., 2003; Morrison et al., 2012; Castillo Ramirez and Urcuqui-Inchima, 2015). This response is started by the physical interaction of the viral molecules with the host-pathogen recognition receptors (PRR) (van Boxel-Dezaire et al., 2006; Basler and García-Sastre, 2007; Fernandez-Garcia et al., 2009). Then, the recognition triggers a signaling pathway that activates transcription factors which induce the expression of IFN-α/β, which bind to specific receptor on the cell surface triggering Jak-STAT antiviral signaling pathway (van Boxel-Dezaire et al., 2006; Basler and García-Sastre, 2007). However, several studies have demonstrated that the flavivirus NS2A, NS2B-3, NS4A, NS4B, and NS5 proteins inhibit type I IFN response (Muñoz-Jordán et al., 2003, 2005; Fredericksen and Gale, 2006; Ashour et al., 2009; Mazzon et al., 2009; Laurent-Rolle et al., 2010; Rodriguez-Madoz et al., 2010). Moreover, the flaviviruses hijack cellular elements in the ER membranes to induce the formation of their cholesterol-rich RC (Mackenzie et al., 2007; Rothwell et al., 2009; Soto-Acosta et al., 2013) where the recognition of viral component for the PRR is avoided, inhibiting IFN response until the replication process is completed (Welsch et al., 2009; Gillespie et al., 2010; Uchida et al., 2014; Miorin et al., 2016).

On the other hand, the key components of the IFN signal transduction pathway are the STAT proteins (STAT1 and 2), which are found in the cholesterol-rich microdomains, lipid rafts (Sehgal et al., 2002; Shah et al., 2002; Marchetti et al., 2006). Interestingly, the flaviviral infection induces the biosynthesis and redistribution of the cellular cholesterol to the RC also leads to the disruption of the cholesterol-dependent surfaces domains founds in the plasma membrane at later times of infection, inactivating the IFN-regulated Jak-Stat signaling pathway (Mackenzie et al., 2007) (Figure 1). This pathway is also inactivated directly by the non-structural proteins as described before (Muñoz-Jordán et al., 2003, 2005; Fredericksen and Gale, 2006; Ashour et al., 2009; Mazzon et al., 2009; Laurent-Rolle et al., 2010; Rodriguez-Madoz et al., 2010). Additionally, it is important to note that there is a close correlation between cholesterol and the type I IFNs response during viral infections because high cholesterol levels induce a poor IFN response (Liu et al., 2013). Thus, the reduction of cholesterol levels during flavivirus infection could guarantee an increase in the response of the IFN.

## Cholesterol-lowering therapy as potential therapeutics in flaviviral infections

Arbovirus infections, especially those caused by flaviviruses such as DENV, ZIKV, WNV, and YFV, represent an immense global health problem (Boldescu et al., 2017). Therefore, it is urgent to find broad-spectrum agents that provide an opportunity to treat these infections. Since there are no specific drugs to treat the flaviviral diseases and aspects such as high genetic variability (Lim et al., 2013), antibody-dependent enhancement (ADE), and cross-reactivity between flaviviruses, make difficult to obtain a fully effective vaccine against all flavivirus (Villar et al., 2015; Halstead and Russell, 2016). The traditional approach for antiviral design is to target critical viral factors. However, this strategy in RNA viruses which do not have a proofreading function leads to a high rate of mutation in the virions, which induce the development of resistance to the antiviral drugs (Acosta and Bartenschlager, 2016; Boldescu et al., 2017). The other possibility is to target host factors. The genus Flavivirus belonging to the Flaviviridae family, which includes DENV, ZIKV, and WNV differs in many aspects, such as the mode of transmission or the course of the infection. However, the fundamental replication strategy of the members of the family is similar, as it requires the formation of organelle-like structures defined as RC, where replication of the genome of positive-strand RNA [(+) RNA] viruses occur in close association with cellular endomembranes (Neufeldt et al., 2018). The RC formation requires among other components, cholesterol (Welsch et al., 2009; Acosta and Bartenschlager, 2016; Neufeldt et al., 2018). Changes in serum cholesterol have been correlated with clinical manifestations in DENV infected patients (van Gorp et al., 2002; Suvarna and Rane, 2009; Biswas et al., 2015; Durán et al., 2015; Osuna-Ramos et al., 2018). Therefore, the development of therapeutic strategies to restrict biosynthesis or cholesterol absorption with FDA-approved drugs, like statins, is an attractive option with broad-spectrum activity possibilities. Statins are molecules of fungal origin that inhibit a crucial step in the biosynthetic pathway of sterol (inhibitors of the HMGCR) (Sirtori, 2014). Statins are powerful cholesterol-lowering drugs used in the therapy of dyslipidemias and have contributed significantly to the prevention of cardiovascular disease (Sirtori, 2014). There are several studies on the use of statins in DENV infection, ranging from cellular to preclinical and clinical trials where opposite effects have been observed (Martínez-Gutierrez et al., 2011, 2014; Whitehorn et al., 2016). For example, *in vivo* models of infection and treatment found that the use of Lovastatin inhibits DENV RNA replication and viral secretion in primary cultures of human monocytes and other cell lines (Rothwell et al., 2009; Martínez-Gutierrez et al., 2011, 2014; Soto-Acosta et al., 2013; Bryan-Marrugo et al., 2016). In the AG129 mouse model, permissive for DENV infection, lovastatin treatment resulted in a 2-day delay in virus-induced mortality, which was independent of the time point at which treatment was initiated (Martínez-Gutierrez et al., 2014). The delay in mortality in the mice model did not correlate with a reduction in viral RNA levels. Considering that statins are widely used and well-tolerated, they could be candidates for a prophylactic emergency antiviral regimen in DENV infections (Boldescu et al., 2017). However, lovastatin treatment was unable to inhibit DENV infection in a clinical trial in humans (Whitehorn et al., 2016). Although it is well known that in humans statins modify systemic lipid levels, the real effect on cellular cholesterol content is unclear. Thus, it is possible that the inability of the statins to inhibit DENV infection could be related with only a partial reduction in cellular cholesterol content, which could be rapidly compensated.

On the other hand, there is an indirect way to induce inactivation of the HMGCR enzyme, using metformin, a drug commonly prescribed to treat patients with type 2 diabetes (Crofford, 1995). This drug increases the activity of the AMPK, the sensor of cellular energy, resulting in the inhibition of HMGCR activity and thus to the suppression of cholesterol biosynthesis (Kawaguchi et al., 2002; Foretz and Viollet, 2011; Koren-Gluzer et al., 2013). Interestingly, in recent work by Soto-Acosta et al. (2017) a significant inhibition in DENV replication was observed in cells treated with metformin, supporting its antiviral activity. Moreover, the use of other AMPK activators such as NDGA or 6-choloro-5-[4-(1-hydroxycyclobutyl) phenyl]-1*H*-indole-3-carboxylic acid (PF-06409577), also induces a strong inhibition in WNV, ZIKV, and DENV infection (Soto-Acosta et al., 2014; Merino-Ramos et al., 2017; Jiménez de Oya et al., 2018). Interestingly, in a retrospective cohort study of adult diabetics patients with DENV infection treated with metformin had a ~37% lower risk of developing severe dengue (Htun et al., 2018), suggesting that metformin has a potential protective effect against dengue disease.

Another compound related with the inhibition of cholesterol metabolism is that related with the blockage of enzyme 7-dehydrocholesterol-Δ7-reductase that performs the final conversion step of 7-dehydrocholesterol to cholesterol, by AY-9944 (Mackenzie et al., 2007), also inhibit flavivirus replication. Moreover, other compounds as the okadaic acid that modulate the of PP2A phosphatase activity (Soto-Acosta et al., 2017) and the hymeglusin and zaragozic acid A which inhibit the HMGCR synthase and squalene synthetase, respectively (Rothwell et al., 2009), display an anti-flaviviral effect. In the same line of evidence, the treatment with 25-hydroxycholesterol (25-HC) which decreases the amount of SREBP-1 (Wang et al., 1994), a factor that enhances transcription of the LDLr and HMGCR genes, inhibits the flaviviral infection (Mackenzie et al., 2007), and reduces the viremia providing protection against ZIKV in mice and rhesus macaques (Li et al., 2017). Additionally, the disruption of the lipid raft formation by MβCD that deplete cellular cholesterol; the cholesterol chelation with filipin (Lee et al., 2008); and the decrease of the fatty acid biogenesis by cerulenin and C75 (Heaton et al., 2010) induce viral inhibition at entry and replication levels. In the case of the C75 drug also decreases the number of lipid droplets affecting the production of the viral particles (Samsa et al., 2009) and the amphipathic steroid 3-β-[2-(diethylamino)ethoxy]androst-5-en-17-one (U18666A) that reduces the cholesterol biosynthesis and also block the intracellular trafficking of cholesterol (Poh et al., 2012), decreases DENV infection.

Further analysis in animal models and clinical trials is essential to determine whether the use of drugs such as other statins, metformin or other AMPK activators in single-dose or in combination as a treatment against flavivirus infections is feasible.

Since the increase in cholesterol levels during flavivirus infection is the consequence of an increase in cholesterol synthesis and an increase in cholesterol uptake through the LDL receptors (Soto-Acosta et al., 2013), it may be possible to inhibit viral infection not only inhibiting synthesis but also decreasing uptake. Recent evidence suggests that pre-treatment with bovine lactoferrin inhibits infection of all four DENV serotypes in Vero cells blocking the binding of DENV to the cell membrane by interaction with heparan sulfate, DC-SIGN, and LDLr (Chen et al., 2017). Bovine lactoferrin might inhibit infection with DENV or other flaviviruses by binding to potential receptors, such as LDLr. Another receptor for cholesterol uptake that can be pharmacologically blocked by the FDA-approved drug ezetimibe is the Niemann-Pick C1 Like 1 NPC1L1 (Garcia-Calvo et al., 2005; Ge et al., 2008). Ezetimibe is a potent inhibitor of cholesterol absorption that has been approved for the treatment of hypercholesterolemia. Rodent livers express only a negligible amount of NPC1L1 (Altmann et al., 2004). However, humans have detectable levels of NPC1L1 in the liver cells (Betters and Yu, 2010). The liver is an important target organ in flavivirus infection (Thepparit et al., 2004). Thus, ezetimibe could be considered a suitable candidate against infections caused by flavivirus (DENV, ZIKV, YFV, or WNV), just as it already had against HCV (Sainz et al., 2012) and hepatitis B virus (HBV) (Lucifora et al., 2013). Since viral infection induces an increase in cholesterol uptake and synthesis to support flavivirus replication (Rothwell et al., 2009; Poh et al., 2012; Soto-Acosta et al., 2013, 2014), a combination of drugs, which inhibit uptake and synthesis, may induce a more efficient HDA therapy for flavivirus inhibition.

## Concluding remarks

Viral infections transmitted by mosquitoes, such as those caused by the flaviviruses DENV, YFV, WNV, and ZIKV represent essential health challenges. The presence of the four DENV serotypes in many parts of the world, the propagation of the mosquito vector and the climate conditions for mosquito proliferation increase the risk to contract severe dengue. On the other hand, the association between ZIKV infection with microcephaly in neonates and with Guillain-Barré syndrome in adults, in addition to its ability to be transmitted through sexual and transplacental routes, made it a threat to global health. Finally, although YFV was confined mainly to monkeys, outbreaks in humans in Angola and Brazil have generated an alarm. Although in Brazil part of the population is immunized and new vaccination programs are in progress, in the rest of America and the world, most of the human population is not immunized. These facts highlight the importance of the study of flaviviruses and the development of new control strategies. It is well-known that every step of the viral life cycle, from entry to viral release requires close interaction with cellular proteins. Therefore, viruses hijack cellular proteins, components and processes to be replicated in the host cell. One of these components is the cellular cholesterol, required for the formation of RC, present in the ER. The reduction in the cholesterol levels with FDA-approved drugs that inhibit cholesterol synthesis or uptake *in vitro* and *in vivo* models induce a reduction of infection with DENV and ZIKV among others. Additionally, the close interaction between cholesterol synthesis and IFN response pathway indicate that the reduction in cholesterol levels could also increase IFN response inhibiting viral infection. Thus, cholesterol can be considered an excellent target to inhibit flavivirus infection. The use of FDA-approved cholesterol-lowering drugs alone or in combination can be tested in new clinical trials.

## Author contributions

JO-R, JR-R, and RdA preparing manuscript, writing, correction, and figures design. JR-R and JO-R performed infection and performed microscopy assays.

### Conflict of interest statement

The authors declare that the research was conducted in the absence of any commercial or financial relationships that could be construed as a potential conflict of interest.
